# Mesothelioma and asbestosis in a young woman following occupational asbestos exposure: Short latency and long survival: Case Report

**DOI:** 10.1186/1746-1596-5-81

**Published:** 2010-12-16

**Authors:** Enrique Bitchatchi, Klaus Kayser, Marina Perelman, Elihu D Richter

**Affiliations:** 1 Unit of Occupational and Environmental Medicine, Braun School of Public Health and Community Medicine, Faculty of Medicine, Hebrew University, Jerusalem, Israel; 2 Telepathology Consultation Center at the Institute of Pathology Charité Medical Faculty of the Humboldt-University, Berlin, Germany; 3 Department of Pathology, Sheba Medical Center, Tel Hashomer, Israel

## Abstract

A 27-year-old female white-collar worker was diagnosed in 1998 with mesothelioma eight and one-half years following first exposure as a bystander to debris in a site in which asbestos-containing building materials were being dismantled and rebuilding work took place. Prodromal back pain had been present for a year and a half. She underwent extrapleural pneumectomy and received an intrapleural infusion of cisplatin post-operatively. Exposure to asbestos was verified by contemporary reports and lung biopsy, which demonstrated asbestos bodies and microscopic interstitial fibrosis -conforming evidence for asbestosis. The patient is alive and well 12 years after diagnosis and 14 years after onset of symptoms. The combination of an extremely short latency period and long survival following occupational exposure to asbestos dust is unique.

## Case Presentation

**Figure 1:**
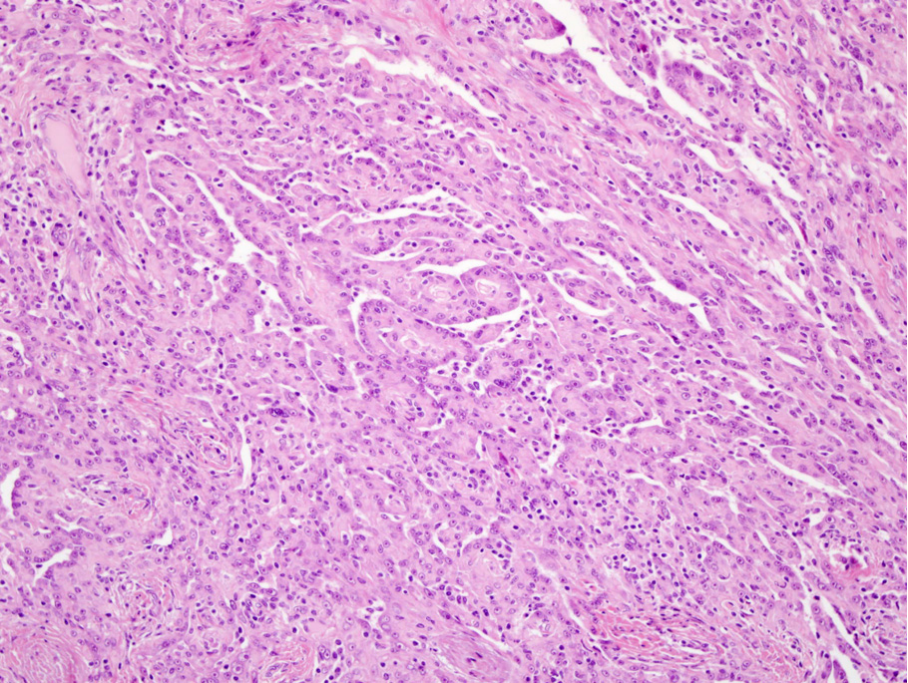
**Malignant mesothelioma, epithelioid variant**. The tumor exhibits papillary and tubular pattern (H&E, original magnification × 200)

**Figure 2:**
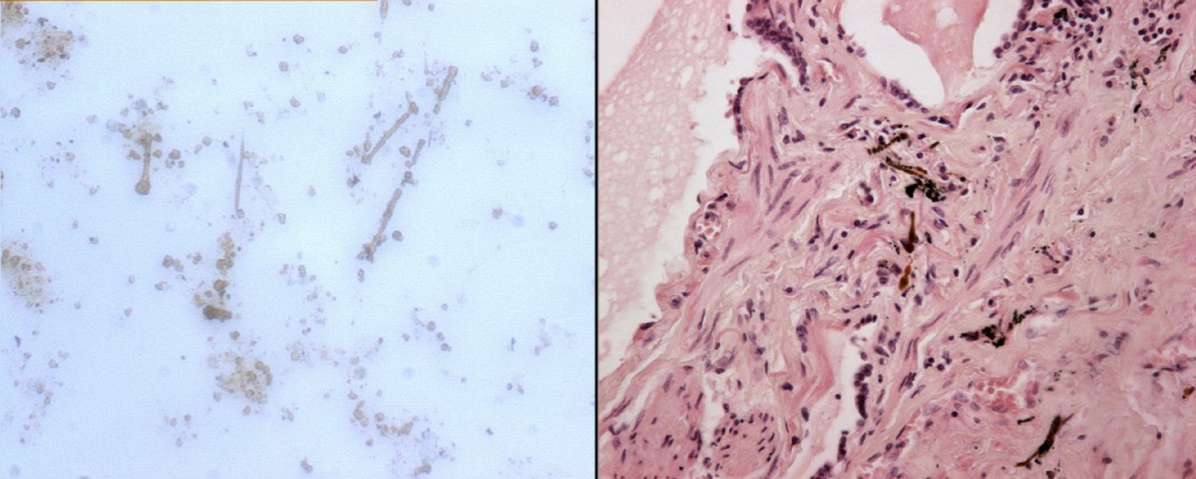
**Asbestos bodies (l) and lung tissue with asbestos bodies (r)**.

A 27-year-old Israeli born woman presented with upper back pain and shortness of breath, diagnosed as Tietze's syndrome in 1996. The pain radiated to her right flank and impeded rest. Chest radiography performed 6 months following presentation was negative. In the 18 months following onset of complaints the patient underwent examinations at orthopedic and pain clinics, including spine (D6-L2) computed tomography (CT), and physical therapy, but showed no improvement. The tomography, performed nine months after onset of symptoms, revealed a thickening process anterior to D9. Three months later, chest radiography showed minimal interstitial changes including few peripheral small opacities on the lower right field. These signs were overlooked. The left lung was clear and showed no pleural or parenchymal abnormalities. Forced expiratory volume in one second (FEV_1_) and forced vital capacity (FVC) were 95% and 96% the predicted value, respectively. Fourteen months after the onset of symptoms, she began losing weight and appetite. CT of the abdomen and chest and focused magnetic resonance imaging showed right pleural thickening and 10 mm focal ipsilateral lung nodules. Tissue biopsy from transpleural thoracoscopy and subsequent complete right extrapleural pneumonectomy indicated mesothelioma and right after surgery, she received an intrapleural infusion of cisplatin. Stage was T1b N0 M0 (according to International Mesothelioma Interest Group 1995). No calcified pleural plaques were found. Three years following surgery her best FVC was recorded as 53% the expected value. Five years after diagnosis, a cardiopulmonary function test indicated restrictive postpneumonectomy pattern, excellent functional compensation and preserved cardiac reserve; however, the test (aimed at 15 Watts/min performance) was interrupted due to dyspnea, which was attributed to suboptimal endurance. Fourteen years from presentation, the patient is in remission, she is working and has completed a successful pregnancy. Histological examination indicated tubopapillary type mesothelioma -Figure 1- (verified by the US-Canadian Mesothelioma Panel- formal correspondence exchange with oncology institute; signed by Panel's chairman in 1997, Dr. A. Churg). Scattered bronchioles showed mild fibrosis without extension into adjacent alveoli. The degree of fibrosis was classified as grade 1 (severity- 1; extent-1) according to NIOSH/CAP criteria for pathologic grading of asbestosis [1]. Quantitative light microscopic count of cytospinned asbestos bodies extracted by wet digestion yielded 305 asbestos bodies/g wet lung tissue, 19 times our reference value [2,3] (Figure 2).

## Exposure history

The patient recalled her first exposure to dusts during intensive demolition/construction work at her workplace, an airport office. The work--the first job in her life, when drafted into the military-- began in 1989 when she was 20 years of age, and continued for approximately 6 years. Co-workers under judicial investigation recalled seeing asbestos wallboard and debris at the site. Her work routine included excessive work hours, sleeping on site and irregular work shifts (approximately 5000 hours of passive, intermittent, exposure to airborne dust). During the 4^th ^calendar year, she became pregnant and got post labor permission for 3 months. No measurements of concentrations of asbestos, constituents of environmental tobacco smoke, man-made-vitreous-fibers and respirable particulate, dusts or gasses were carried out. Information was not available on the mix of fibers in the dusts during demolition at her work site. However, a newspaper article in 1989, contemporary with exposure, reported that Dr Joseph Ribak, then chief of occupational medicine of the major national health care provider, specified that her work site was one of several with acoustic ceilings sprayed with asbestos fibers [4]. We also know that in Israel, the fiber mix of most asbestos construction products was approximately 90% chrysotile -10% amphiboles [5].

The patient's past exposure history was otherwise unremarkable, with negative answers to directed questions on exposures as bystanders to asbestos or talc in family, work, home, or hobbies. Her father worked as a police officer and her mother as a housewife, partially self-employed in sewing jobs. No other working adult lived in the household. Based on an official list of all her past addresses, we double-checked those obtained by detailed anamnesis confirming no history of residency near asbestos cement plants or brake lining plants from infancy onward. She denied having contact with Thorotrast.

## Discussion

To the best of our knowledge, the seven-year latency period between first exposure and complaints, and 8.5 years (14 months lapsed between symptoms and diagnosis) to diagnosis of mesothelioma is the shortest ever reported in an adult. The case for this conclusion rests on both tissular *and *independent ascertainment of exposure, notably the contemporary report by occupational health authorities specifically referring to asbestos dust exposures at the patient's workplace. The light microscopy technique, with a 60× objective lens is too low a magnification to detect the vast majority of asbestos fibers retained. The moderately elevated concentration above background -in the patient's resected lung is supportive of her past work exposures causing mesothelioma [6-13], given the absence of any other known source. The finding of asbestosis is a strong confirmation that she had substantial asbestos exposure.

We note that the demonstration of several asbestos bodies in a light micrograph of lung tissue when combined with interstitial fibrosis on histology is sufficient also for a diagnosis of asbestosis using standard NIOSH/College of American Pathology criteria [1]. Her unilateral imaging features are not in agreement with pulmonary fibrosis as set by current criteria. However, histologically proved asbestosis has been recognized elsewhere, despite lack of abnormalities on a CT scan [14]. Our case represents indeed a deviant from common acceptance of an incubation of tens of years to diagnosis of this pneumoconiosis [15,16], with a minimum latency of 10 years [17]. The occupational history and the discovery of asbestos facts in conjunction with the histopathologic detection of interstitial fibrosis make the hypothesis for a "spontaneous case" for mesothelioma most unlikely.

Davis and Rall published a table, which specified that the latent period for cancer in persons with workplace exposures to high volume carcinogens can range from 4 to 40 years [18]. A study from Poland reported 16 cases of pleural mesothelioma found among a cohort exposed from 1987 to 1997. Four of the patients were employed for periods ranging from 3.5 months to five years. Two of the four had latency periods of 11-12 years from onset of occupational exposure. These four patients had occupational and prior residential exposures associated with massive use of commonly available asbestos-cement wastes as road surface material [19]. Our report along with that from Poland calls into question the general consensus that latencies for mesothelioma under 10 years are improbable.

An absence of calcified pleural plaques in our patient may imply shorter latency since exposure [20,21]. Animal experiments with implantation of asbestos or other fibers in the pleura or peritoneum show that the latent period shortens as fiber dose is increased and lengthens as the dose of fiber is reduced [20]. The present report indicates that high prior exposures, particularly at younger ages, may result in shortened induction periods, in keeping with classic observations on increased dose and shortened latency [22-24].

Individuals with direct exposures associated with the construction trades are apt to be the most heavily exposed [25]. During pregnancy, the physiological augmentation of minute ventilation leads to a greater dust burden into lung parenchyma. As with ionizing radiation, the earlier the age at exposure, the shorter the latency period for asbestos related cancers [26]. Worker cohort studies indicate that earlier age of exposure predicts incremental lifelong adjusted risks for mesothelioma [27,28] and persons near asbestos work are at risk of "bystander exposure" [29,30].

In comparison with mixed and sarcomatoid, the epithelial cell type described in our patient, predicts better prognosis [31,32].

## Conclusions

This patient was diagnosed with asbestosis and mesothelioma eight and one-half years following asbestos exposure independently reported at the time of its occurrence. She has survived twelve years post diagnosis and resection. Although unexpected longevity has been reported elsewhere [33,34], we have no explanation for the long survival despite the very short latency.

## Consent

Written informed consent was obtained from the patient for publication of this case report and any accompanying images. A copy of the written consent is available for review by the Editor-in-Chief of this journal.

## Abbreviations

NIOSH: National Institute for Occupational Health and Safety; CAP: College of American Pathologists; Other abbreviations were defined in the text.

## Competing interests

The authors declare they have no competing interests, whether financial or non-financial. Leading author (EB) did act as expert witness appointed by the patient. The litigation ended in 2003. There will be no reopening of the judicial process.

## Authors' contributions

EB saw the patient 4 years after the pneumonectomy, then carried out the clinical and epidemiologic investigation and wrote most of the drafts. KK carried out the fiber counts in his laboratory; he performed histological analysis including asbestosis grading. MP contributed pursuing further histological examinations and revisions as to set asbestosis classification as well. ED revised and helped rewrite the drafts and added material to the discussion. All authors read and approved the final manuscript.

## References

[B1] Craighead JE Abraham JL Churg A Green FH Kleinerman J Pratt PC Seemayer TA Vallyathan V Weill H The pathology of asbestos-associated diseases of the lungs and pleural cavities: diagnostic criteria and proposed grading schema. Report of the Pneumoconiosis Committee of the College of American Pathologists and the National Institute for Occupational Safety and Health Arch Pathol Lab Med 1982 106 544 96 6897166

[B2] Kayser K Becker C Seeberg N Gabius HJ Quantitation of asbestos and asbestos-like fibers in human lung tissue by hot and wet ashing, and the significance of their presence for survival of lung carcinoma and mesothelioma patients Lung Cancer 1999 24 89 98 10.1016/S0169-5002(99)00035-5 10444059

[B3] Kayser K Seemann C Andre S Kugler C Becker C Dong X Kaltner H Gabius HJ Association of concentration of asbestos and asbestos-like fibers with the patient's survival and the binding capacity of lung parenchyma to galectin-1 and natural alpha-galactoside- and alpha-mannoside-binding immunoglobulin G subfractions from human serum Pathol Res Pract 2000 196 81 87 10707363 10.1016/s0344-0338(00)80037-0

[B4] Valentin A End of asbestos era 1989 Haaretz, Weekend Magazine 12

[B5] Richter ED Asbestos exposure in Israel: Findings, issues and needs Israel J Med Sci 1984 20 89 97 6368466

[B6] Gylseth B Mowe G Skaug V Wannag A Inorganic fibers in lung tissue from patients with pleural plaques or malignant mesothelioma Scand J Work Environ Health 1981 7 109 13 7313614 10.5271/sjweh.2559

[B7] Howel D Gibbs A Arblaster L Swinburne L Schweiger M Renvoize E Hatton P Pooley F Mineral fiber analysis and routes of exposure to asbestos in the development of mesothelioma in an English region Occup Environ Med 1999 56 51 8 10.1136/oem.56.1.51 10341747 PMC1757652

[B8] Karjalainen A Nurminen M Vanhala E Vainio H Anttila S Pulmonary asbestos bodies and asbestos fibers as indicators of exposure Scand J Work Environ Health 1996 22 34 8 8685671 10.5271/sjweh.106

[B9] McDonald JC Armstrong BG Edwards CW Gibbs AR Lloyd HM Pooley FD Case-referent survey of young adults with mesothelioma: lung fiber analysis Ann Occup Hyg 2001 45 513 8 11583653

[B10] Mowe G Gylseth B Hartveit F Skaug V Occupational asbestos exposure, lung-fiber concentration and latency time in malignant mesothelioma Scand J Work Environ Health 1984 10 293 8 6523093 10.5271/sjweh.2326

[B11] Pooley FD Berry G Rogers AJ Mesothelioma - asbestos exposure and lung burden Sci Publication Issued IARC Lyon 1989 90 486 96 2545618

[B12] Rodelsperger K Jockel KH Pohlabeln H Romer W Woitowitz HJ Asbestos and man-made vitreous fibers as risk factors for diffuse malignant mesothelioma: results from a German hospital-based case-control study Am J Ind Med 2001 39 262 75 10.1002/1097-0274(200103)39:3<262::AID-AJIM1014>3.0.CO;2-R 11241559

[B13] Srebro SH Roggli VL Samsa GP Malignant mesothelioma associated with low pulmonary tissue asbestos burdens: a light and scanning electron microscopic analysis of 18 cases Mod Pathol 1995 8 614 21 8532693

[B14] Paris C Benichou J Raffaelli C Genevois A Fournier L Menard G Factors associated with early-stage pulmonary fibrosis as determined by high resolution computer tomography among persons occupationally exposed to asbestos Sand J Work Environ Health 2004 30 206 214 10.5271/sjweh.781 15250649

[B15] Balmes J LaDou J Occupational Lung Diseases Current Occupational and Environmental Medicine 2007 4 New York: McGraw-Hill 310 327

[B16] Lanphear BP Buncher CR Latent period for malignant mesothelioma of occupational origin J Occup Med 1992 34 718 21 1494965

[B17] Chahinian AP Pass HI Kufe DW, Weichselbaum RR, Pollock RE, Basts Jr RC, Gansler TS, Holland JF, Frei E Malignant Mesothelioma Cancer Medicine 2006 American Cancer Society: BC Decker Hamilton 1447 61

[B18] Davis DL Rall DP Lorenz K, Ng Y, Davis DL Risk Assessment for Disease Prevention Strategies for Public Health 1981 Van Nostrand, Reinholt [The table is cited in pp. 258-260, Davis DL, Secret History of the War on Cancer. New York: Basic Books; 2007:505]

[B19] Szeszenia-Dabrowska N Wilczynska U Szymczak W Laskowicz K Environmental exposure to asbestos in asbestos cement workers: a case of additional exposure from indiscriminate use of industrial wastes Int J Occup Med Environ Health 1998 11 171 7 9753896

[B20] Parkes WR Occupational lung disorders 1994 3 Oxford: Butterworth-Heinemann

[B21] Becklake MR Occupational lung disease-past record and future trend using the asbestos case as an example Clinical and Investigative Medicine 1983 6 305 317 6671361

[B22] Armenian HK Lilienfeld AM Distribution of incubation periods of neoplastic diseases Am J Epidemiol 1974 99 92 100 4359273 10.1093/oxfordjournals.aje.a121599

[B23] Smith PG Doll R Mortality among patients with ankylosing spondylitis after a single treatment course with X-rays Br Med J (Clin Res Ed) 1982 284 449 460 10.1136/bmj.284.6314.449 6800494 PMC1496076

[B24] Whittemore AS Age distribution of human cancer from carcinogenic exposure of various intensities Am J Epidemiol 1977 106 418 432 920729 10.1093/oxfordjournals.aje.a112484

[B25] Agency for Toxic Substances and Disease Registry (US) Case studies in environmental medicine 1990 Department of Health and Human Services. San Rafael, California --- Either first page or author must be supplied.

[B26] Hall JC DeVita VT, Helman S Principles of Carcinogenesis Cancer: Principles and Practice of Oncology 1993 4 Rosenberg SA: Philadelphia: Lippincott 213 227

[B27] National Research Council Scientific Report 1984 Washington. D.C.: National Academy Press

[B28] Peto J Doll R Hermon C Binns W Clayton R Relationship of mortality to measures of environmental asbestos pollution in an asbestos textile factory Ann Occup Hyg 1985 29 305 355 10.1093/annhyg/29.3.305 4073702

[B29] Ringen K Englund A Segal JL McCann M Lemen RA Levy BS, Wegman DH Construction Workers Occupational Health: Recognizing and preventing work-related disease and injury 2001 Philadelphia: Lippincott, Williams & Wilkins 756

[B30] Oliver LC Rom WN Asbestos in Public Buildings Environmental and Occupational Medicine 1992 2 Boston: Little, Brown and Company 293 300

[B31] Robinson BWS Musk AW Lake RA Malignant mesothelioma TheLancet.com 2005 366 397 408 10.1016/S0140-6736(05)67025-0 16054941

[B32] Becklake MR Bagatin E Neder JA Asbestos-related diseases of the lungs and pleura: uses, trends and management over the last century Int J Tuberc Lung Dis 2007 11 356 369 17394680

[B33] Fischbein A Suzuky Y Selikoff IJ Bekesi JG Unexpected longevity of a patient with malignant pleural mesothelioma: report of a case Cancer 1978 42 1999 2000 10.1002/1097-0142(197810)42:4<1999::AID-CNCR2820420447>3.0.CO;2-Y 309354

[B34] Neuman V Müller KM Fischer M Malignant Mesothelioma-German mesothelioma register 1987-1999 Int Arch Occup Environ Health 2001 74 383 395 10.1007/s004200100240 11563601

